# IL-17 mRNA in sputum of asthmatic patients: linking T cell driven inflammation and granulocytic influx?

**DOI:** 10.1186/1465-9921-7-135

**Published:** 2006-11-03

**Authors:** Dominique MA Bullens, Els Truyen, Liesbeth Coteur, Ellen Dilissen, Peter W Hellings, Lieven J Dupont, Jan L Ceuppens

**Affiliations:** 1 Department of Experimental Medicine, Clinical Immunology, Katholieke Universiteit Leuven, (KULeuven), Leuven, Belgium; 2 Department of Woman and Child, Paediatrics, KULeuven, Leuven, Belgium; 3 Department of Internal Medicine, Pneumology, KULeuven, Leuven, Belgium; 4 Department of Head and Neck Surgery, Otorhinolaryngology, KULeuven, Leuven, Belgium; 5 Recipient of a postdoctoral fellowship from the Fund for Scientific Research (FWO), Vlaanderen; 6 Recipient of a senior clinical research fellowship from FWO, Vlaanderen

## Abstract

**Background:**

The role of Th2 cells (producing interleukin (IL-)4, IL-5 and IL-13) in allergic asthma is well-defined. A distinct proinflammatory T cell lineage has recently been identified, called Th_17 _cells, producing IL-17A, a cytokine that induces CXCL8 (IL-8) and recruits neutrophils. Neutrophilic infiltration in the airways is prominent in severe asthma exacerbations and may contribute to airway gland hypersecretion, bronchial hyper-reactivity and airway wall remodelling in asthma.

**Aim:**

to study the production of IL-17 in asthmatic airways at the mRNA level, and to correlate this with IL-8 mRNA, neutrophilic inflammation and asthma severity.

**Methods:**

We obtained airway cells by sputum induction from healthy individuals (n = 15) and from asthmatic patients (n = 39). Neutrophils were counted on cytospins and IL-17A and IL-8 mRNA expression was quantified by real-time RT-PCR (n = 11 controls and 33 asthmatics).

**Results:**

Sputum IL-17A and IL-8 mRNA levels are significantly elevated in asthma patients compared to healthy controls. IL-17 mRNA levels are significantly correlated with CD3γ mRNA levels in asthmatic patients and mRNA levels of IL-17A and IL-8 correlated with each other and with sputum neutrophil counts. High sputum IL-8 and IL-17A mRNA levels were also found in moderate-to-severe (persistent) asthmatics on inhaled steroid treatment.

**Conclusion:**

The data suggest that Th_17 _cell infiltration in asthmatic airways links T cell activity with neutrophilic inflammation in asthma.

## Background

Asthma is a T cell driven chronic inflammatory disorder of the airways [1]. Both T helper (Th)2 and Th1 lymphocytes, play an important role in the pathophysiology of asthma [2-4]. Local overproduction of T helper (Th)2 cytokines (IL-4, IL-5, IL-9 and IL-13) by Th2 cells in the asthmatic airways is well defined [4-6] and recent studies indicate that Th1 cells, secreting IFN-γ, might cause severe airway inflammation [7-9]. Recently, a separate T cell lineage, called Th_17 _cells or inflammatory T cells, producing IL-17A (or IL-17), has been identified [10,11]. Th_17 _cells might potentially play an important role in the pathophysiology of asthma. IL-17 is especially important for the recruitment of neutrophils [12] and is expressed in bronchial biopsies, bronchoalveolar lavage fluid and sputum of patients with asthma [13-15]. A role for IL-17 in murine asthma models has also been described [16,17] and overexpression of IL-17 in lung epithelium causes chemokine production and leukocyte infiltration *in vivo *[10]. Neutrophils are especially prominent in acute, severe exacerbations of asthma [18,19]. Moreover, it has been suggested that at least two inflammatory subtypes of asthma exist: the eosinophilic and the non-eosinophilic type [20]. The subgroup of patients with neutrophilic asthma is characterized by poor response to corticosteroids [21]. Neutrophils potentially contribute to airway gland hypersecretion, bronchial hyper-reactivity and to airway wall remodelling [22,23] by producing matrix metalloproteinase-9 (MMP-9) observed in broncho-alveolar lavage fluid from moderate-to-severe asthma patients [24].

The mechanism of neutrophil recruitment in asthma is still unclear, but several chemokines may have a role [25]. Among those, CXCL8 (IL-8), secreted by T lymphocytes, epithelial cells, smooth muscle cells and macrophages might especially be important, as CXCL8 (IL-8) is increased in the airways of patients with asthma [26-29].

The non-invasive technique of sputum induction has been developed to obtain viable cells from the lower airways of asthma patients and healthy individuals [30] and was later shown to be useful for studies on cytokine mRNA expression by semi-quantitative [31] and quantitative RT-PCR techniques. With this technique, mRNA levels for cytokines and chemokines are normalized to a house-keeping gene in order to correct for differences in the cell number and amount of cDNA amongst the different samples studied [32]. This method therefore avoids some of the problems associated with protein measurements in sputum samples. We have recently described the use of this method for the quantification of Th1/Th2 cytokines at the mRNA level in induced sputum [9]. To further study a potential role of Th_17 _cells in asthma, we used the technique of induced sputum in combination with quantitative real-time RT-PCR to quantify the expression of IL-17A and IL-8 mRNA in relation to each other, to CD3γ mRNA as a quantification of T cells, and to the neutrophilic inflammation in the airways of patients with asthma.

## Materials and methods

### Subjects

This study was performed between September 2002 and August 2004. Subjects have been extensively described elsewhere [9]. In brief, thirty-nine asthmatic subjects (16 women, 23 men), not taking systemic steroids and 15 age-matched healthy controls (8 women, 7 men) between 18 and 65 years were recruited. Asthma severity was scored on the basis of the Global Initiative for Asthma (GINA) criteria [33]. Mild intermittent (n = 11) and mild persistent (n = 12) asthma patients were grouped as mild asthmatics, and moderate (n = 8) and severe (n = 8) asthmatics were grouped as moderate-to-severe asthmatics. Asthmatic subjects were further subdivided into atopics (n = 21) and non-atopics, (n = 17) based on detection of specific IgE antibodies for house-dust-mite (n = 19), pets (n = 12) and/or pollen (grass or tree) (n = 17) and on a clinical history suggestive of allergic responses to those allergens. No differences in FEV1% (Fishers' exact test: p = 0.48), asthma severity (GINA classification) (Fishers' exact test: p = 0.49) and inhaled corticosteroids (ICS) use (Fishers' exact test: p = 0.28) between the allergic and the non-allergic asthmatics were found.

Patients were allowed to continue their usual treatment. Thirteen patients regularly used ICS: <500 μg/day Beclomethason Dipropionate (BDP) or equivalent (n = 1), 500–1000 μg/day BDP or equivalent (n = 5) and >1000 μg/day BDP or equivalent (n = 7); "non-users" either had never used ICS or had not used them since at least 3 months. A significantly larger proportion of patients with moderate-to-severe asthma used ICS in comparison to patients with mild asthma (Fishers' exact test: p < 0.0001).

Symptoms were measured using the asthma symptom scores (ASS) [34] and the Asthma Control Questionnaire developed by Juniper of which a validated Dutch translation is available [35]. Those scores significantly correlated with each other [9]. The FEV1 was measured by means of a spirometry. Bronchial hyperresponsiveness was determined by measuring the histamine concentration that provoked a 20% decrease in FEV1 (PC20). Exhaled nitric oxide (NO) was measured with an Ecophysics CLD 700 AL MED chemiluminescence analyser (n = 16)(Dürnten, Switzerland).

Healthy controls had normal spirometry, no present clinical symptoms of upper or lower airway disease, ASS of zero and they did not use presently or in the past 5 years any anti-asthma medication. Five of the 15 healthy controls had allergic rhinoconjunctivitis and two others had a history of oral allergy symptoms [9]. Sputum was not collected during the months April, May, June and July which correspond to the tree-pollen and grass-pollen season in Belgium.

The study was approved by the local ethical committee of the Faculty of Medicine, Leuven.

### Sputum induction and analysis

Sputum was induced by inhaling increasing concentrations of hypertonic saline (3%, 4% and 5% for 7 minutes) generated by a De Vilbiss Nebulizer (Ultra-NebTm 2000 model 200HI) after pre-treatment with 400 μg of inhaled salbutamol (unless a fall in FEV1 of greater than 10% occurred, in which case the procedure was stopped).

Sputum was processed by a modification of the technique described by Pizzichini *et al *[36] which was recently published [9]. In brief, all sputum plugs that appeared free of salivary contamination were selected. Sputum was treated by adding a volume of Hanks' Balanced Salt Solution containing 0.1% dithiothreitol (Sigma, St. Louis, MO, USA) and 3% Bovine Serum Albumin (Sigma). The cell pellet was resuspended in 1000 μl RPMI 1640 (Bio Whittaker Europe, Cambrex) containing 2 mM L-glutamine, penicillin (100 U/ml), streptomycin (100 μg/ml) (Bio Whittaker Europe) and 10% bovine calf serum (BCS) (Hyclone, Logan, UT, USA). Sputum squamous cell percentage varied between 0% and 35.6% (mean 6.6%). The cell suspension was adjusted to 1.0 × 10^6 ^cells/ml, and 50,000 cells were put in a Shandon 3 Cytocentrifuge (Techgen, Zellik, Belgium). Cytospins were air-dried and stained using May Grünwald Giemsa. In each sample, 250 leukocytes were counted and the percentage of each specific cell type was determined.

### Measuring cytokine mRNA

Cells were stocked on lysis buffer from the Qiagen Mini Rneasy kit (Maryland, USA) at -20°C until use. RNA was isolated from induced sputum of 33 patients and 11 control subjects with the Qiagen Mini Rneasy kit (Maryland, USA). Both differential cell count and cytokine mRNA were measured in 29 of the patients. RNA was transcribed to cDNA with the Ready-to-go T-primed First Strand Kit (Amersham Pharmacia biotech, Uppsala, Sweden). Real-time quantitative PCR was performed for interleukin (IL)-8, IL-17A, IL-5, CD3γ and β-actin in the ABI prism 7700 Sequence Detector System (Applied Biosystems, Foster City, CA) as described [32]. cDNA plasmid standards, consisting of purified plasmid DNA specific for each individual target, were used to quantify the target gene in the unknown samples, as described [32]. All results were normalised to β-actin to compensate for differences in the amount of cDNA. For each cytokine at least one primer or probe spans an intron. The primer and probe sequences for IL-17A and CD3γ were designed with Primer Express (Applied Biosystems):

IL-17A FW 5'AATCTCCACCGCAATGAGGA3'

IL-17A RV 5'ACGTTCCCATCAGCGTTGA3'

IL-17A TP 5'FAM-CGGCACTTTGCCTCCCAGATCACA-TAMRA3'.

CD3γ FW 5'TCATTGCTGGACAGGATGGA3'

CD3γ RV 5'GGGCTGGTAGAGCTGGTCATT3'

CD3γ TP 5'FAM-CGCCAGTCGAGAGCTTCAGACAAGC-TAMRA3'

IL-8 primers and probe were a gift from Prof. Dr. G. Verleden (division of Pneumology, UZ Gasthuisberg). The primers and probes for IL-5 and IL-8 have been reported [9,37]. All primers and probes were purchased from Applied Biosystems or Eurogentec S.A.

### Statistics

Statistical analyses were performed with GraphPad Prism (GraphPad Software Inc., San Diego, USA) by using the non-parametric Kruskall-Wallis, Mann-Whitney U test or student-t test were appropriate. Variances were tested with the F test and normality was analysed with the Kolmogorov Smirnov test. Correlation studies were performed by Spearman non-parametric test. Contingency tables were analysed by Fisher's exact test. A difference was considered to be significant when p < 0.05.

## Results

**Figure 1:**
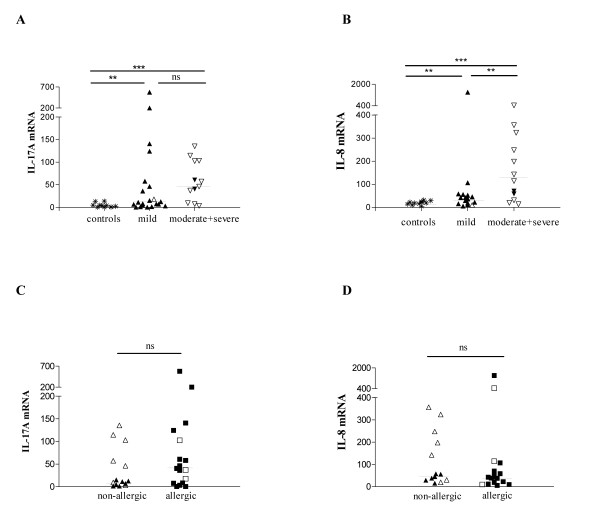
**IL-17A and IL-8 mRNA levels in healthy controls and asthmatics**. RNA was isolated from induced sputum of healthy controls (n = 11) and asthmatic patients (n = 33) and real time RT-PCR was performed with β-actin, IL-17A and IL-8 specific primers and with VIC-(β-actin) or FAM-(IL-17A, IL-8) labelled specific probes. Results were quantified by the use of a cDNA plasmid-standard and expressed as the ratio of cDNA copy numbers for IL-17A (A-C) or IL-8 (B-D) divided by the cDNA copy numbers for β-actin multiplied by 10^4 ^(A-C) or 10^1 ^(B-D). Patients were divided in subgroups according to asthma severity following the reviewed GINA criteria [33] (A, B) and allergic state (allergic state was not known in one patient and two patients with only specific IgE for grass pollen were excluded for the comparison of allergic to non-allergic asthma) (C, D). Open symbols represent patients treated with corticosteroids, closed symbols represent patients without corticosteroid-treatment. Comparisons were performed using the Kruskall-Wallis and Mann-Whitney-U test. The median is indicated with a horizontal line. * = p < 0.05, ** = p < 0.01, *** = p < 0.001 and ns = not significant.

Sputum samples were obtained from 15 controls and 39 asthmatics. Sputum mRNA expression of IL-17A (figure 1A) and IL-8 (figure 1B) was significantly higher in asthmatic patients in comparison to healthy controls. Increased IL-17A mRNA and/or IL-8 mRNA levels could discriminate asthma patients from the healthy control group at a cut-off of 5 for IL-17A mRNA (p = 0.0006 by Fisher's exact test) and at a cut-off of 30 for IL-8 mRNA (p = 0.0009 by Fisher's exact test).

Asthma severity was evaluated on the basis of the revised Global Initiative for Asthma (GINA) criteria [33]. When compared to healthy control individuals, IL-17A and IL-8 mRNA levels in induced sputum were significantly higher in mild asthmatics and in moderate-to-severe asthmatics (figure 1A,B). IL-8 mRNA levels were higher in patients with moderate-to-severe asthma than in patients with mild asthma, whereas IL-17A mRNA levels were similarly elevated in both subgroups (figure 1A,B). A significantly higher proportion of patients with moderate-to-severe asthma then with mild asthma had very high levels of IL-8 mRNA (above 50) and/or IL-17A mRNA values (above 50) (IL-8 mRNA: cut-off >50, p = 0.009 and IL-17A mRNA cut-off >50: p = 0.03 by Fisher's exact test respectively).

**Figure 2:**
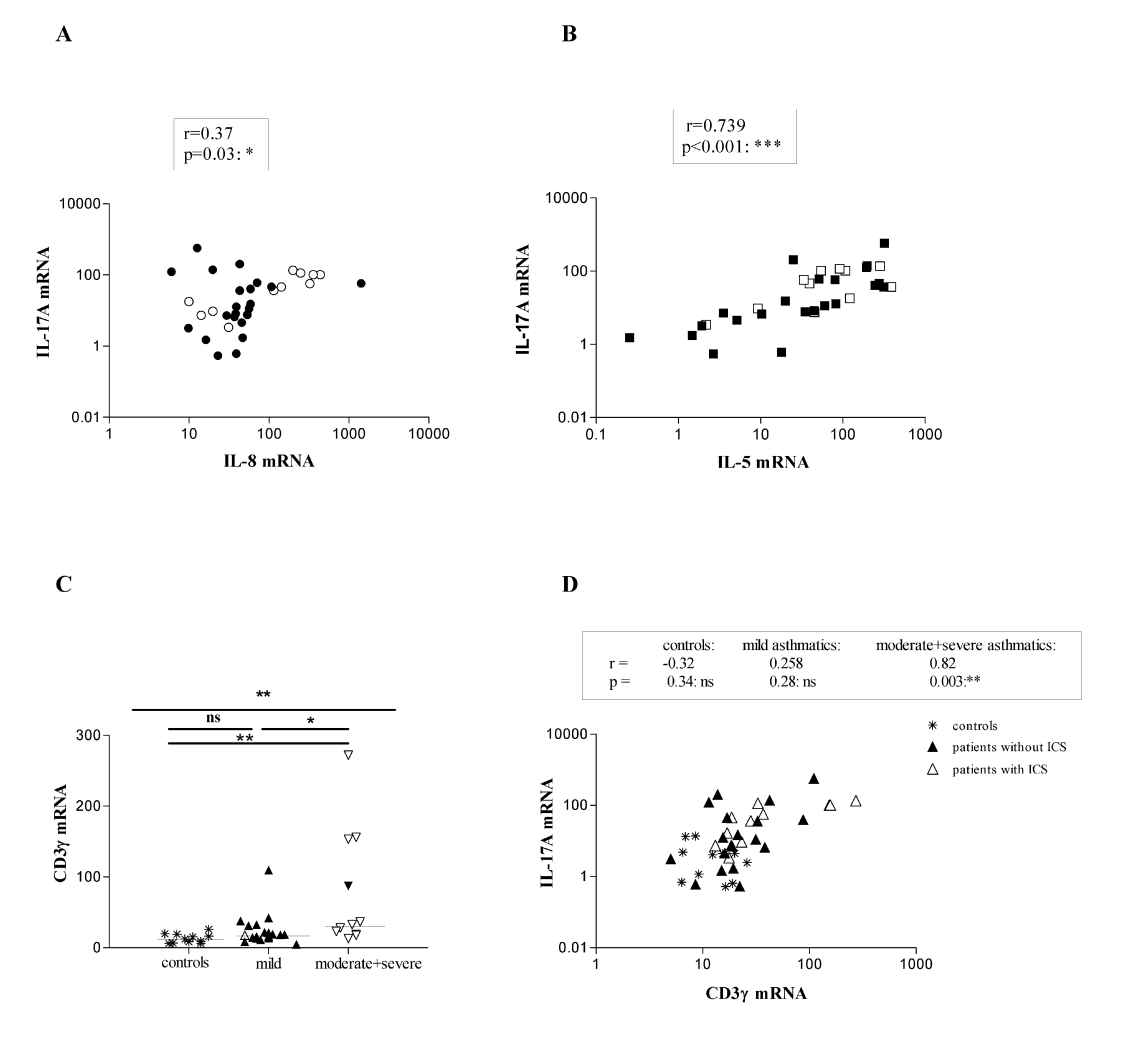
**CD3γ mRNA levels in healthy controls and asthmatics, correlations between IL-17A and CD3γ and between IL-17A other cytokine/chemokine mRNA levels**. CD3γ, IL-8, IL-17A and IL-5 mRNA levels in induced sputum of asthmatic patients were quantified as explained in figure 1 using IL-8, IL-17A and IL-5 specific primers and VIC-(β-actin) or FAM-(IL-8, IL-17A, IL-5) labeled specific probes. Asthma severity was determined as in figure 1A. Open symbols represent patients treated with corticosteroids, closed symbols represent patients without corticosteroid-treatment. Comparisons were performed using the Kruskall-Wallis test and Dunn's post test (C). Correlations were studied by Spearman non-parametric test (A-B, D). The median is indicated with a horizontal line. * = p < 0.05, ** = p < 0.01, *** = p < 0.001, and ns = not significant.

Although patients treated with corticosteroids (open symbols in figure 1B) tended to have higher levels of IL-8 mRNA in comparison to those that did not receive corticosteroids (closed symbols), the difference between those two groups was not statistically significant. IL-17A and IL-8 mRNA expression were similarly distributed in non-allergic and allergic patients (Figure 1C,D). As shown in figure 2A, the IL-17A and IL-8 mRNA levels correlated significantly with each other, which was expected as IL-17A has been shown to induce IL-8 release [25]. However, and to our surprise, the IL-17A mRNA levels also correlated significantly with the IL-5 mRNA levels (figure 2B). Data for IL-5 were separately reported [9].

We found no correlation between the asthma symptom control on the basis of the scoring systems and the IL-17A and/or IL-8 mRNA levels, whether studied in all patients or in the steroid-naive patients only (data not shown). NO in exhaled air [38] and airway hyper-responsiveness measured by histamine provocation (PC20) did not correlate with either IL-17A or IL-8 mRNA, whether steroid naïve and steroid treated patients were studied separately in two subgroups or as one group (data not shown).

**Figure 3:**
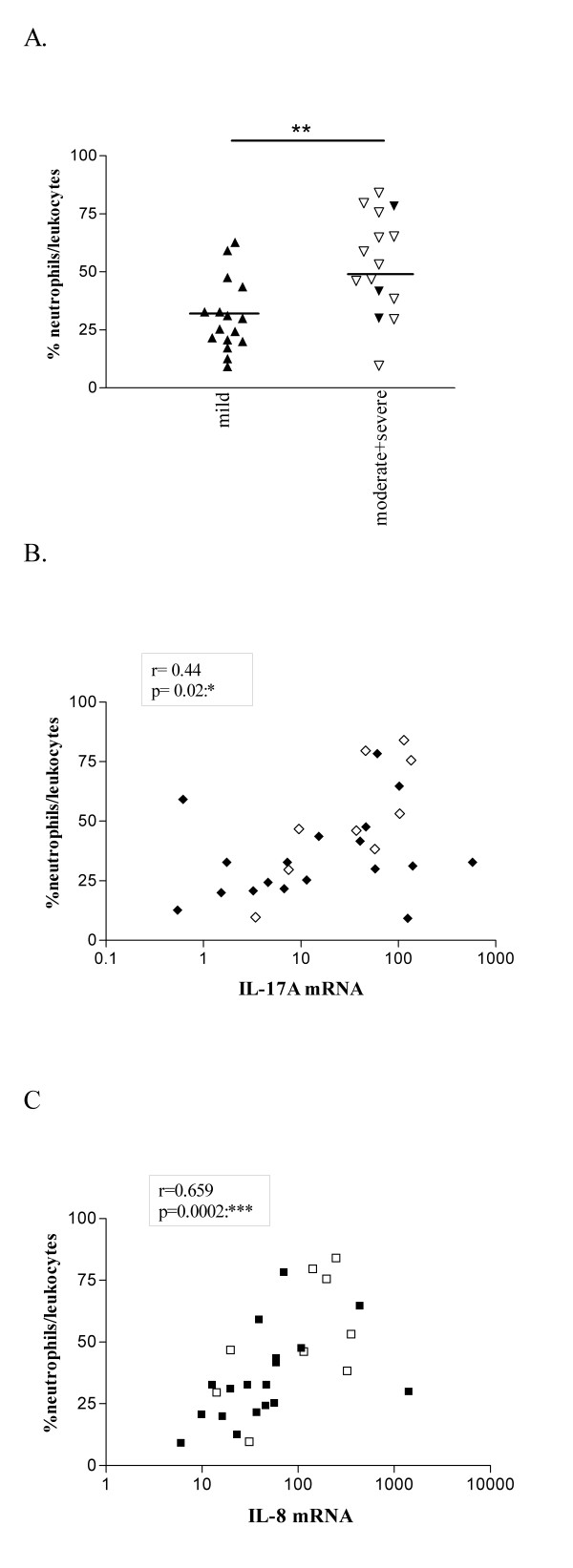
**Sputum neutrophil counts in healthy controls and asthma patients and correlation of IL-17A and IL-8 mRNA levels with the neutrophil count in asthmatics**. Cytospins of induced sputum of healthy controls (n = 11) and asthmatic patients (n = 32) were stained with May Grünwald Giemsa. Neutrophils are counted as percentage of the leukocytes. IL-17A and IL-8 mRNA levels in induced sputum of asthmatic patients (n = 27) were determined as explained in figure 1 and 2. Open symbols represent patients treated with corticosteroids, closed symbols represent patients without corticosteroid-treatment. The mean is indicated with a horizontal line. Differences are studied by student t-test (A) and correlation was studied by Spearman non-parametric test (B-C). * = p < 0.05, ** = p < 0.01 and *** = p < 0.001.

Levels of CD3γ mRNA, reflecting infiltrating T cells are low in controls and significantly increased in moderate-to-severe asthmatics (figure 2C). In the asthmatic patient group in total (r = 0.5, p = 0.0079) and in the subgroup of moderate to severe asthmatics (r = 0.8, p = 0.003), a significant positive correlation between CD3γ mRNA and IL-17A mRNA levels was observed (figure 2D). Compared to mild asthmatics, increased sputum neutrophil counts were observed amongst patients with moderate-to-severe asthma (figure 3A, open symbols represent patients on inhaled steroids). Neutrophil counts were significantly higher in steroid-treated than in steroid-naïve patients (p < 0.05, data not shown). As shown in figure 3B–C, both IL-17A and IL-8 mRNA levels significantly correlated with the sputum neutrophil (p = 0.02, r = 0.4 and p = 0.0002, r = 0.7 respectively) but not eosinophil (p = 0.4, r = 0.2 and p = 0.7, r = -0.09 respectively)(data not shown) count in the patient group. In the subgroup of steroid-naïve patients, IL-8 mRNA levels also significantly correlated with the sputum neutrophil count (p = 0.003, r = 0.7) but IL-17A mRNA levels did not (p = 0.2, r = 0.3).

We found no correlation between the sputum neutrophil count and the ASS or the ACQ (data not shown). Allergic and non-allergic asthmatics had comparable sputum neutrophil counts (data not shown).

## Discussion

In this study we have investigated local IL-17A, IL-8 and CD3γ mRNA expression in relation to the cellular infiltration in the airway lumen from asthmatic patients and healthy controls as assessed by sputum induction. We found higher levels of both IL-17A and CD3γ (reflecting T cell infiltration) mRNA in asthmatic patients when compared to healthy controls. Levels of CD3γ were highest in the moderate and severe patient group and correlated in that group with IL-17A mRNA expression, suggesting increased levels of the newly described Th_17 _cell lineage [10,11]. This correlation was absent in healthy controls and even tended to be negative, showing that T cells in the airways of healthy individuals do not belong to the Th_17 _cell lineage. Moreover, all healthy individuals had low to undetectable IL-17A and IL-8 mRNA levels in their sputum cells, which makes increased IL-17A or IL-8 mRNA levels potentially useful as a diagnostic aid in asthma. We also found in the asthmatic population that both IL-17A and IL-8 mRNA levels correlate significantly with the sputum neutrophil count. This correlation was recently also observed by other authors at the protein level [14]. A significant correlation between IL-8 mRNA and IL-17A mRNA on the one hand and with neutrophils on the other hand suggests (although it does not provide proof for) cause-effect relationship. Human bronchial epithelial cells, fibroblasts and airway smooth muscle cells recruit neutrophils into the airways by the release of CXCL8 (IL-8) and/or other chemokines (such as granulocyte chemotactic protein-2 and growth-related oncogene(GRO)-α) upon stimulation by IL-17[13,39,40]. Until now, no other cellular source for IL-17 besides T cells has been identified. IL-17 in the airways is most likely produced by a T helper lineage called 'inflammatory T cells' or Th_17 _cells [10,11] and this could represent the link between T cell inflammation and granulocytic influx. The study of those 'inflammatory T cells' might become of specific interest in order to unravel differences between eosinophilic and non-eosinophilic asthma phenotypes [20,41]. Somewhat surprising, the levels of IL-17 and IL-5 mRNA positively correlated with each other. This suggests that the inflammatory cell type and the Th2 type are both recruited in inflamed airways. Another explanation could be that both cytokines can be secreted by the same cell type. This is the aim of further study.

In order to study whether different subgroups of asthma patients could have differences in the expression of sputum IL-17A or IL-8 mRNA levels, we compared allergic to non-allergic asthmatics and mild to moderate/severe asthmatics. Allergic and non-allergic patients had similar IL-17A and IL-8 mRNA levels, but differences in the IL-17A and IL-8 mRNA expression level depending on disease severity could be found. High IL-17A and/or IL-8 mRNA levels were always found in patients with moderate/severe asthma while only approximately half of the mild asthmatics had increased IL-17A and/or IL-8 mRNA levels in their sputum. In accordance with this, the highest sputum neutrophil count was also found in the subgroup of patients with moderate-to-severe asthma, as also observed by other authors [14,25]. Most of the moderate-to-severe asthmatic patients were treated by inhaled corticosteroids. Our findings are consistent with recent observations suggesting that corticosteroid treatment can increase the number of neutrophils in endoscopic biopsy specimens from patients with moderate-to-severe asthma [42]. Increased presence of neutrophils as observed in patients treated with inhaled corticosteroids might thus reflect enhanced neutrophil survival upon treatment with steroids, rather than higher disease severity. This might be due to the fact that corticosteroids protect neutrophils against apoptosis while they induce eosinophil apoptosis [43]. Another potential explanation is that neutrophil recruiting Th_17 _cells or cytokines are steroid resistant. A significant limitation is that it is clinically challenging to correlate sputum IL-17A mRNA expression with neutrophil influx (and disease severity) while excluding the confounding variable of inhaled corticosteroid use, which could independently contribute to neutrophil influx. To resolve this discussion, IL-17A and IL-8 mRNA expression as well as neutrophil influx should now be studied before and after the introduction of inhaled steroids. The persistence of IL-17 and of neutrophils despite steroid treatment is indeed of potential prognostic significance as neutrophils can contribute to the phenomenon of airway remodeling and irreversible airway obstruction [13,22].

In conclusion our findings suggest Th_17 _T cell infiltration and ensuring production of IL-17A mRNA in asthmatic airways in parallel with increased IL-8 mRNA levels as the plausible cause of neutrophilic infiltration. High IL-17A and/or IL-8 mRNA levels were found in patients with moderate-to-severe asthma, even if those patients were treated with corticosteroids. In those patients, high IL-17A levels correlate with CD3γ expression, suggesting that a substantial proportion of airway T cells are Th_17 _cells. These might be responsible for neutrophil recruitment, with IL-8 as an important intermediate chemokine.

## Abbreviations

ASS: asthma symptom score

BDP: Beclomethason Dipropionate

ICS: inhaled corticosteroids

eNO: exhaled nitric oxide

FEV1: forced expiratory volume in 1 second

IFN-: interferon

IL: interleukin

mAb: monoclonal antibody

mRNA: messenger RNA

PC20: histamine dose resulting in a 20% decrease in FEV1

Th: T helper

## Competing interests

The author(s) declare that they have no competing interests.

## Authors' contributions

DMA Bullens participated in study design, was responsible for primers and probes design, performed statistical analysis and drafted the manuscript. E Truyen and L Coteur both carried out the sputum inductions, sputum examinations, differential cell counts, cDNA preparations and performed patient questionnaires. E Dilissen carried out the RT-PCR analysis. P Hellings participated in study design and coordination and also in interpretation of the data. L Dupont and J Ceuppens were both responsible for study design, patient recruitment and data interpretation. All authors read and approved the final manuscript.
